# Nurturing care: perceptions and practices of caregivers for children under five in the Ecuadorian highlands – a qualitative study

**DOI:** 10.3389/fpubh.2024.1373896

**Published:** 2024-11-11

**Authors:** Betzabé Tello, Maria J. Mendoza-Gordillo, Marcelo Moreano, Benjamin R. Bates, Katherine Quinn, Camila Rogel, Mario J. Grijalva

**Affiliations:** ^1^Center for Research in Health in Latin America (CISeAL), Pontifical Catholic University of Ecuador, Quito, Ecuador; ^2^Facultad de Medicina, Pontifical Catholic University of Ecuador, Quito, Ecuador; ^3^Infectious and Tropical Disease Institute (ITDI), Department of Biomedical Sciences, Heritage College of Osteopathic Medicine, Ohio University, Athens, OH, United States; ^4^School of Communication Studies, Scripps College of Communication, Ohio University, Athens, OH, United States; ^5^Facultad de Ciencias Exactas y Naturales, Pontifical Catholic University of Ecuador, Quito, Ecuador

**Keywords:** child care, caregivers, parenting, child development, nurturing care framework

## Abstract

**Introduction:**

The importance of nurturing care for child development is well-established, and parents play a central role in providing this care. However, cultural values and traditions can influence child-rearing practices, and there are gaps in child welfare in Ecuador. Two research questions delve into caregivers’ definitions of nurturing care for children aged 0–5 and its alignment with World Health Organization’s concept.

**Methods:**

A qualitative methodology was applied to comprehensively explore caregivers’ perspectives and application of nurturing care across diverse cultural contexts in rural areas of Cotopaxi and Loja in Ecuador. Using snowball sampling primary caregivers, healthcare professionals, childcare workers, and community leaders were interviewed and participated in focus groups, examining its congruence with WHO’s Nurturing Care Framework for Early Childhood Development.

**Results:**

While there is alignment with the WHO framework, the study reveals challenges such as a lack of awareness of the term “nurturing care” among participants. Findings also indicate issues in health communication, reliance on traditional medicine, and myths around nutritional practices. The role of technology in early learning is explored, noting both its advantages and disadvantages. Notably, preventive health activities were not mentioned, emphasizing a universal need for knowledge.

**Conclusion:**

This study urges tailored interventions for nurturing care, emphasizing success tied to robust healthcare and child protection. Urgency lies in cultural sensitivity, local adaptation, and targeted training for implementation. These insights contribute significantly to the global discourse, stressing the importance of context-specific approaches. Implications are crucial for policymakers, practitioners, and researchers dedicated to elevating care quality for vulnerable populations worldwide.

## 1 Introduction

Scientific evidence spanning the last three decades has consistently underscored the profound significance of establishing a robust foundation for healthy development within the first thousand days of a child’s life, commencing from conception to age two (1). This period emerges as pivotal in shaping a child’s future behavioral patterns, learning capabilities, and overall health. Parents play a central role in this process, fostering emotional and cognitive development for the child, while also mitigating stress and shielding against adverse effects (1). Responsive caregiving during this time supports rapid brain development, influencing the structure and capacity of the brain in ways that can have long-term benefits (2). The WHO’s Nurturing Care Framework is a comprehensive guide designed to promote early childhood development by integrating five essential components: good health, adequate nutrition, responsive caregiving, security and safety, and opportunities for early learning (3). These interconnected elements aim to ensure that children not only survive but thrive in their development, providing a holistic approach that considers the well-being of both children and caregivers. The framework encourages governments and organizations to develop policies and programs that support these components, emphasizing the need for integrated action across the health, nutrition, education, and social protection sectors (2).

Nurturing care supports the rights of children to grow into healthy adults. The importance and fulfillment of economic, cultural, social, protection, and political rights for the children of the world is clear. These rights are well established and accepted by the member states in the United Nations Convention on the Rights of Children (4). Although all children have these rights, cultural values and traditions exert a pronounced influence on child-rearing practices, and these practices profoundly affect child development (5, 6). Within a given society, cultural scripts serve as guiding principles for parents, shaping their beliefs and practices concerning child development. Not all cultural scripts are supportive of nurturing care. Importantly, it is crucial to acknowledge that variations can exist within a culture, contingent upon developmental levels, temperament, and gender (5, 6).

The findings from the 2018 National Health and Nutrition Survey in Ecuador highlight issues related to nurturing care for children. Key indicators depict significant challenges: 40% of children under five live in impoverished households, and 23% experience stunting. Access to basic services, including safe water, sanitation, and hygiene, is incomplete, especially in rural areas. Limited access to children’s books at home adds to the challenges, and only about 6% of fathers engage in stimulating play. Alarmingly, 50% of children under five experience mistreatments at home, and neglect is common, with 7.1% of children cared for by another child under 10. In summary, nearly 28% of Ecuadorian children under five face inadequate safety and limited opportunities for holistic development (7).

Recognizing the pivotal role of nurturing care in child well-being and acknowledging the gaps in child welfare in Ecuador, our study delves into the perceptions and practices of caregivers responsible for children under 5 years of age in the Ecuadorian highlands, specifically within rural communities in Cotopaxi and Loja communities. Our focus is on understanding how nurturing care is conceptualized and practiced. To formalize our inquiry, we pose the following research questions:

RQ1: How do caregivers of children aged 0–5 define nurturing care?

RQ2: Do these definitions align with the concept of nurturing care as endorsed by the WHO?

Addressing these questions is crucial for informing the development and implementation of programs and interventions aimed at fostering nurturing environments. This, in turn, contributes to ensuring the optimal growth and development of children within these distinctive contexts.

## 2 Materials and methods

### 2.1 Design

This research employed a qualitative methodology to comprehend and explore the understanding and practices regarding nurturing care of primary caregivers, healthcare professionals, childcare workers and community leaders. Interviews and focus groups were conducted with caregivers living in rural areas of Ecuador. The interviews and focus groups were transcribed and thematized to answer RQ1. These themes were then compared to recommendations from existing literature to answer RQ2. This methodology allowed us to understand the practices of the specific caregivers and to contextualize those lived practices within broader literature, thereby respecting the voices of both caregivers and experts in the field of child development.

### 2.2 Setting

Data collection for this study was conducted from May to July 2023 in rural areas of two Andean provinces in Ecuador: Loja and Cotopaxi. Although both provinces are situated in the Andean mountains, they possess distinct contextual factors associated with child development challenges, as highlighted in previous research. In Loja, bordering Peru in the southern Ecuadorian Andes, poverty and agricultural labor contribute to child underdevelopment. Here, we engaged caregivers in three rural communities in Calvas county, Chile parish: Chaquizhca, Guara, and Bellamaria. Nearly all (98.5%) of residents are mestizo, with most families practicing subsistence farming or day labor.

In contrast, our second location was in Cotopaxi, a central Andean province where we engaged seven rural communities in Sigchos county, Chugchilán parish: Pueblo, Chazualó, Guanto, Chinaló Bajo, Chinaló Alto, Sector Uno, Itopungo. These communities are predominantly indigenous (95.4%) and rely heavily on agriculture and livestock production. Notably, indigenous children in Ecuador experience higher rates of underdevelopment (8).

The fieldwork in both provinces revealed high poverty rates and significant challenges in child development, such as stunting and limited access to services. These realities illustrate how cultural practices and socioeconomic conditions shape child-rearing in rural Ecuador. By examining the nurturing care practices of Loja’s mestizo population and Cotopaxi’s Indigenous communities, the study provides valuable insights into the diverse cultural and economic contexts affecting child development in these rural settings.

### 2.3 Participants

To be invited to participate, caregivers had to live with children under 5 years of age and reside in one of the communities of interest. Other key informants, such as childcare workers, healthcare workers, and community leaders, had to reside in the communities of interest, but may have had children under 5 years of age or not. Participants need to have reached the age of majority (9) or be emancipated teenage caregivers (considered to have legal capacity for civil acts in Ecuador). Any individual who did not meet these criteria, did not speak Spanish, or who had a mental disability that would not allow them to provide informed consent were excluded.

The researchers announced the study in churches, stores, and health facilities, and through community leaders, allowing potential participants to establish contact with the research team. Prospective participants were encouraged to share information about the study with acquaintances who might meet the research’s inclusion criteria.

Upon contacting the researchers, prospective participants were provided with informed consent materials. If prospective participants signed the consent form and agreed to participate, they were informed of the date and time for the focus groups. These focus group sessions lasted approximately 60 to 90 min, while individual interviews lasted around 60 min.

Before the interviews and focus groups, the research team explained the informed consent process, stating that they were researchers from the Pontificia Universidad Católica del Ecuador and Ohio University, trained in health sciences. They emphasized their goal of collaborating with the community to understand the challenges of child-rearing for children under five. Throughout the interview and focus group process, the researchers emphasized that they were there to learn from the community members. The research team intentionally did not define what nurturing care was to the participants to allow the community members’ perspectives to drive the conversation. This introduction fostered trust and openness, allowing participants to share their experiences and facilitating the data collection process.

The study involved a diverse group of participants. The participants were categorized into three groups: Primary caregivers (parents), Community Leaders, and Healthcare/Childcare workers belonging to the establishments of Ministry of Health (MoH) and Ministry of Economic and Social Inclusion (MIES).

In Ecuador, all doctors must complete a rural medical service year (medicatura rural), providing primary care in underserved rural communities. This program ensures that newly graduated doctors deliver essential healthcare services to these populations, although they often rotate to other positions after completing their service. Healthcare teams, known as Equipos de Atención Integral (EAIS), consist of a physician, a nurse, and a Community Health Technician (TAPS), who is a local resident familiar with community practices and geography. The TAPS remains in the community, enhancing outreach efforts, while the physician and nurse typically change roles after their service year. Similarly, the Ministry of Economic and Social Inclusion (MIES) employs community members in programs like Child Development Centers (CDI) and Growing with our Children (CNH). These programs provide support and home visits for families with children under 3 years old, and most professionals working in these initiatives are also from the communities they serve, deepening their understanding of local challenges and practices.

Twenty-two primary caregivers, specifically parents of children under 5, willingly participated in our interview sessions. Six primary caregivers in Sigchos and 16 in Calvas participated. The sample consisted primarily of mothers (20 participants), with one father and one grandmother included Participants were recruited through a snowball sampling method, where initial participants referred others, allowing for a broader representation of perspectives and experiences in nurturing care. In this referral methodology, although all caregivers were required to have a child under 5 years of age, to diversify the sample we asked seed participants to provide referrals that included diverse socioeconomic status and representation from geographical areas across the Cantons. Furthermore, we conducted 21 interviews involving health workers, childcare workers, and community leaders, with 6 in Sigchos and 15 in Calvas, intentionally seeking a diverse perspective.

Additionally, we organized six focus groups, including primary caregivers (*N* = 1) in Calvas, healthcare workers (*N* = 2) in both Calvas and Sigchos, community leaders (*N* = 1) in Sigchos, and childcare workers (*N* = 2) in Calvas. These focus groups delved into their respective roles in supporting child development. The inclusion of this diverse array of participants allowed us to gain a holistic understanding of nurturing care practices within these Ecuadorian communities.

This study was conducted according to the guidelines laid down in the Declaration of Helsinki. All procedures involving research study participants were approved by the Ethics Committee for Research in Human Subjects of the Pontifical Catholic University of Ecuador, code EO-31-2023, V2. Written informed consent was obtained from all participants.

### 2.4 Data collection procedures

Researchers conducted interviews and focus groups in Spanish, with participants’ consent for audio recording. A semi-structured interview guide was used to facilitate the interviews. The questions were open-ended and allowed flexibility to focus on areas of relevance to the participants. The questions were centered around early childhood development (ECD) (e.g., How do you know if your child has an adequate development?) health and nutrition (e.g., When do you usually take your child to the health center?) (e.g., What kind of foods do you usually offer to your child?) responsive caregiving (eg., How do you calm or comfort a child who is upset or aggressive?), early learning opportunities (e.g., What games and activities do you do with your children to enjoy as a family?), and safety and security (e.g., Who takes care of the child most of the time?). These aspects align with the key components of the Nurturing Care Framework (3). The same questions were used in the focus groups. The study followed a snowball sampling method. We initially identified several healthcare professionals and community members who fulfilled the inclusion criteria. We asked them to circulate the announcement of the study among other colleagues and friends and/or suggest other potential participants that might meet the study’s inclusion criteria (10).

### 2.5 Data analysis procedures

In relation to data analysis, in accordance with Tracy’s (10) suggestions for handling qualitative data, the first coding involved the application of *in vivo* coding. This method entails coding each line of what the participants expressed during the interviews. This methodology is also appropriate for addressing research inquiries on individual interpretations conveyed within the data (11). The initial coding facilitated the second author to establish and comprehend the processes occurring within the gathered data. Unlike other forms of coding, the quality of coding is judged through this iterative process rather than through calculated inter-rater reliability measures; any discrepancies in coding were resolved through discussion. After the first round of initial coding, a total of 154 codes were obtained. We employed a constant comparative approach to review the data and categorize the codes into grouped codes for subsequent analysis. This involved comparing parts of interviews within the same interview, as well as drawing comparisons across various interviews (12). Following the initial coding phase, the second round of coding commenced by identifying patterns within the codes derived from the data through focused coding. Focused coding allowed sub-categories for analysis. We finished the focused coding, resulting in the identification of 30 sub-categories. Following the completion of focused coding, we proceeded with axial coding to reintegrate the data that had been divided throughout the two rounds of initial coding and focused coding. We completed the axial coding process and identified a total of 10 categories. The last phase of coding involved theoretical coding.

In terms of translation, we adopted the methodology employed by Lincoln et al. (13) and Gonzalez and Lincoln (14) for conducting cross-cultural and cross-linguistic studies. Furthermore, the first coding cycles were developed in the participants’ native language- Spanish to ensure the consistency of their stories, but the final coding, themes, and narratives were in English. To systematize the data and initial codes during the first phase of coding, we employed ATLAS.ti 9. However, for following rounds of coding, starting with the second phase of *in vivo* coding up to theoretical coding, we relied on manual coding. Regarding transcription, trained students at the Pontifical Catholic University of Ecuador transcribed the interviews and focus groups. All transcripts were anonymized and were protected by using code numbers.

### 2.6 Research team context

The research team consisted of seven members: four from Quito, the capital of Ecuador, and three from the United States. Although not all researchers were originally from the study communities, four team members had prior experience working in these areas through broader projects. In Loja, they were involved in the “Healthy Living Initiative,” and in Cotopaxi, they participated in a collaboration between Pontificia Universidad Católica del Ecuador and the Decentralized Autonomous Government of Chugchilán to improve the quality of life in communities with populations among the poorest quintiles in the country. This prior work provided valuable local insights, enhancing the team’s understanding of the local and cultural dynamics. The combination of internal and external perspectives enabled a comprehensive exploration of nurturing care practices.

## 3 Results

Twenty-two children’s caregivers, ten community leaders, and eleven childcare and healthcare workers were interviewed; overall, forty-three interviews were conducted in the two territories. Each interview lasted between 15 and 45 min. Additionally, six focus groups were carried out. The focus groups had around 12 participants, the smallest focus group had 9 participants and the largest had 15. One focus group was with children’s caregivers, one with community leaders, and four with childcare and healthcare workers. In an effort to bring the participant’s stories to life, we explore the themes and sub-themes that arose from the analysis. We report the participants’ narratives using a code as a pseudonym. The data analysis resulted in three overarching themes: taking care of children, the children’s health a development, and the necessity of knowledge.

### 3.1 Taking care of children

The theme “taking care of children” focuses on sharing the participants’ meanings about nurturing care. Also, it includes the participants’ knowledge about what nurturing care is and their role within this strategy.

All the participants in the study mentioned that they do not know what nurturing care is as a concept formulated in the public health field. However, their role in the caring for and the development of children was understood. It can be seen in the narrative of a family physician who put it:

*“I do not know what nurturing care is. I’ve never heard that before. But I usually explain [to] the mothers that they have to take care of their babies and give them love and attention. I also mention that they do not have to mistreat them.”*

In the same vein, a nurse said: “*I do not know what nurturing care is. We do not receive training about it. I tell to the mothers that they have to love their children and give adequate food.”* Additionally, during the focus group the healthcare providers agreed that they need more training about nurturing care. They said: *“We need more training about the programs that the MoH has. We want to learn more about nurturing care. For us who work in healthcare centers, it is essential. We need to guide mothers on how to take care of their children.”*

Creciendo con Nuestros Hijos (CNH) is a nationwide family education and home visiting program in Ecuador, managed by MIES, designed to assist vulnerable children aged three and under, along with their caregivers. Weekly group visits in public areas are conducted by CNH childcare workers for families with children aged 2–3, and weekly home visits are carried out by childcare workers associated with CNH for families with children under the age of two.

Although the meaning of nurturing care also was not understood for CNH’s staff, they understand the importance of this program for the children’s development and health. One childcare worker put it: *“For me nurturing care is giving love and attention to children. It also involves playing with the baby and giving them good food.”* Similar to the public healthcare providers, the MIES childcare workers mentioned that they want to learn more about nurturing care. They said: *“We want more training and materials related to nurturing care and nutrition.”*

Similarly, the authorities from both the MoH and MIES are not aware of what nurturing care is. A worker from the MoH mentioned: “*I do not know what nurturing care is. However, I believe that it is related to the rights of a child of being cared and loved.*” Similarly, a worker from MIES said: *“Nurturing care is the right of a child to have proper health and caring. However, I did not listen the specific word. But I would like to learn more and include it in the activities for the CNHs.”*

The children’s caregivers never heard about nurturing care nor received information from the healthcare providers or CNHs. A mother mentioned: “*Nurturing care is love and protection to our baby*.” Similarly, during the focus groups the primary caregivers brought attention to the fact that they do not have proper communication with the healthcare providers. A mother said: *“The doctor or the nurse do not explain the things properly; sometimes, I do not understand them.”* However, they argued that they have better communication with the childcare workers of CNH. A mother mentioned: “*The CNH always come to my house every week. She explains me all the things and activities that she is doing with my child. Also, she reminds me to go to the healthcare center.”*

### 3.2 The children’s health and development

This theme highlights the assets and challenges that the participants encountered when taking actions about the health and development of children in both communities. Several participants felt that inadequate communication and myths regarding food, nutrition, responsive caregiving, and opportunities for early learning are challenges whilst health promotion activities and the home visits from the CNHs are assets.

The healthcare workers argued that children’s caregivers usually bring children to the healthcare center when they are ill but not as a preventive measure. A doctor mentioned: “*The mothers most of the time come the healthcare center when their babies are sick and for vaccination. They never come to check if their children are growing up adequately or to prevent sickness.”* Likewise, during the focus groups the CNHs mentioned that although they insist that the carers take the children to the healthcare center as a preventive measure, the caregivers are not used to doing it. A CNH said:

*“The mothers go to the healthcare center or even the hospital when their children have diarrhea, pain, cough, fever but never to know if they are growing up adequately. I even have to insist them to go for vaccination. I believe that most of the mothers do not come to the healthcare center because they have to wait for long time, so they just wait when their children are sick. When their children are mildly ill, they go to the pharmacy to buy the medicine. The salesperson in the pharmacy just gives the medicine they are not doctors.”*

During the focus groups, very few childcare and healthcare workers mentioned that children’s caregivers in the study went to the healthcare center as a preventive action against sickness. As a nurse said, “*they sometimes come to see if their babies are gaining weight and height adequately. Also, they come for vaccines and micronutrients.”*

Similarly, during the focus groups, all the caregivers agreed that they do not seek attention from healthcare providers to prevent any ill or to have guidance regarding their children’s health and development. A mother mentioned:

*“I go to the healthcare center when my baby is very ill. When she is not so sick, I used to go to the pharmacy just to buy a medicine. Sometimes, I prefer to go to a private clinic because I do not have to wait for long time. Also, I believe that in private clinics the service is better.”*

Also, other children caregivers mentioned that they prefer to seek assistance from traditional medicine, a situation that is very common in rural areas of the country. A mother put it: *“When my son is sick, I usually go to the curandero because when my baby has diarrhea it is because some bad spirits approach to him, and the curandero cleans him.”*

On the other hand, there are several myths regarding child nutrition in Ecuador, specifically complementary feeding. The myths are related to what to eat when to start, and foods that have different nutritious properties. In this study, the children’s caregivers shared diverse experiences regarding complementary feeding. During the focus groups, a mother mentioned:

*“I learned about nutrition with my mother and my mother-in-law. They explained [to] me that I should give my baby soups with vegetables and little bit of chicken. Also, I started the complementary feeding with coladas. In the colada I put different fruits. I do not give my baby heavy foods such as pork or some grains. Those foods are not good.”*

Similarly, another mother said: *“I started the complementary feeding with fruits such as apple, pear, banana but the most important fruit was granadilla. Granadilla juice helps children to talk quickly.”* In the same vein, the myths are extended to a multiple micronutrient powder supplement to prevent anemia and vitamin and mineral deficiencies, called “Chispas.” All of the children’s caregivers in the study argued that Chispas causes either diarrhea or constipation and sometimes they prefer not to give the entire doses of the supplement. A mother mentioned:

“The *CNH and the doctor tell me to give Chispas to my child, but sometimes I just give the half of it because since my baby take it, sometimes she has diarrhea. Also, the doctor tells me to give it in the soup whereas the CNH in a small portion of the food.”*

On the other hand, healthcare professionals, CNHs, and children caregivers mentioned that ultra-processed foods such as chocolate, candy, and sweet beverages are not good for children under five years old. As a mother put it: *“Fast food is bad for children because it has a lot of sugar and fat*.” Similarly, a CNH mentioned*: “Here in our communities, children do not eat ultra-processed foods, especially in rural areas. Sometimes children from the center of the town use to drink sweet beverages and milk with chocolate.”* However, during the focus group the healthcare providers mentioned that although the caregivers know the risk of ultra-processed foods for the children health some caregivers give those to make the child happy. A doctor mentioned: *“We explain [to] mothers not to give to children ultra-processed foods, but the mother used to give them milk with chocolates and cookies to keep them calm and not to cry.”*

Regarding responsive caregiving, CNHs mentioned that they explain to the caregivers the importance of taking care of the children with love and attention. A CNH said: *“I tell the mothers to spend time with their children. Also, I tell them to pay attention while they are eating, talking, and playing.”* Similarly, the healthcare providers mention that they provide support to caregivers in regard with responsive caregiving. A doctor said “I *usually tell the mothers to spend quality time with their children to pay attention to their needs. However, sometimes some mothers do not like to listen [to] advice when upbringing their children.”*

Regarding opportunity for early learning, all the healthcare providers and CNH mentioned that early learning through games is essential for the adequately development of children. The MoH and MIES workers suggested that health promotion activities such as counseling are essential to guarantee an adequate understanding of children caregivers. As a doctor said: “*One to one conversation is essential to guide the mother with an adequate way of taking care of her child. It is better than a group education because each child has their own particularity.”* Similarly, CNHs mentioned that another asset are home visits because they can observe the interaction between the caregiver and the child, and the child feels more comfortable in their space. A CNH said: “*When I do the home visits, I have the chance to see how the mother and the baby interacts, and based on that I provide guidance. Also, I spend all my time to each of the children in my neighborhood.”* Likewise, the caregivers in the focus groups agreed that interaction with their children is essential for the early learning of their babies. As one mother put it:

*“I always play with my baby I show her videos in my phone. The CNH tells me to talk to her explaining [to]her what I’m doing, what she’s eating, and to read to her every night. I do not have a lot of resources to buy toys and books for that reason I use my phone. My girl smile at me we laugh a lot. It is a special moment for us.”*

### 3.3 The necessity of knowledge

This theme addresses the sentiments and perceptions that the participants shared during the interviews and focus groups about the necessity of training and how to put into practice the public policy regarding nurturing care.

All the healthcare professionals and social workers mentioned that there is an urgent need for training regarding nurturing care. A doctor said: *“I would like to have more knowledge to provide a better service to my patients. I could guide them better if I have more knowledge.”* Likewise, a CNH mentioned: “*I feel that I do not have enough materials for training mothers. I would like to know more about nurturing care and improve the quality of life of my children.”*

On the other hand, the stakeholders reported that there is a disconnect between community activities and policy. A leader mentioned: “*We do not have enough technical resources to put into practice the nurturing care strategy, especially in rural areas.”* Another leader said: *“There is not enough budget to reproduce material or to have toys and toolkits for nurturing care. As a result, the CNHs have to manage their activities even with their own money.”*

The stakeholders and leaders do not possess enough understanding of nurturing care as was previously mentioned. As a result, there is no toolkit or clear guidelines on how to approach this strategy in the communities. As a stakeholder from MIES said: “We *do not know how to put the nurturing care concept into practice with the families. We need a guideline and materials.”* Likewise, a doctor said: *“All the training are centralized in the main cities and with the authorities we received the training after months if we are lucky.”*

These three themes reflect the narratives and experiences of children caregivers, workers, and stakeholders regarding nurturing care and highlight the opportunities and challenges identified by the participants regarding necessities for reaching the nurturing care strategy.

## 4 Discussion

The findings of our study align with and pose challenges to the World Health Organization’s (WHO) Nurturing Care Framework. The core principles of the framework, emphasizing health, safety, and responsive care, resonate with participants, especially primary caregivers (1). However, a significant challenge arises from participants’ lack of awareness of the term “Nurturing Care” within public health and programs of child protection and development. Despite incorporating nurturing care practices, the term and formal conceptualization appear unfamiliar to participants, including healthcare professionals and childcare workers. Although the global nurturing care proposal seeks to facilitate the holistic development of children in all contexts, limitations in implementation, especially in rural areas, are evident (5, 6, 15–17). The disconnect between policy and on-the-ground implementation, as expressed by stakeholders, exposes a gap that needs addressing.

In the theme of “Child Health and Development,” our findings align with the WHO framework but reveal specific challenges in insufficient communication, the use of traditional medicine, self-medication due to a lack of access to health services, and behaviors lacking preventive care. The caregivers’ tendency to seek healthcare mainly when a child is ill, rather than as a preventive measure, challenges the anticipatory and preventive aspects of the Nurturing Care model. Preventive topics addressed by the Nurturing Care model, such as family planning, avoiding toxic substances at home, HIV transmission prevention, the mental health of children and caregivers, special care for premature and underweight children, and screening for hearing visual, and developmental issues, were not named (2). This highlights the gap in operationalizing global frameworks in diverse sociocultural contexts, emphasizing the importance of considering local beliefs and practices in healthcare interventions.

Additionally, the study identifies myths and misconceptions, especially regarding complementary feeding and the use of nutritional supplements, which can challenge certain aspects of existing nutritional models (18). These community-specific beliefs should be considered in the design of interventions and policies (9). Similarly, while the study strengthens concepts related to child malnutrition, it does not identify preventive measures for overweight and obesity (19).

In our Ecuadorian study, healthcare providers and community health workers emphasized the crucial role of early learning, focusing on interactive methods, particularly through games. They highlighted the importance of individual conversations and home visits for effective guidance and personalized care. While this approach, based on real-time observations and interactions, was deemed valuable for addressing the unique needs of both caregivers and children, primary caregivers mentioned using screen devices to stimulate their children, which can have advantages and disadvantages (20). Exposure to screen-based media can impact socio-emotional development, and recommendations emphasize joint viewing and interaction with caregivers during screen time, requiring a personalized media plan to select suitable content and create usage plans, especially in resource-limited settings. The central recommendation for children’s screen use is to restrict screen time for those under 2 years (except for video chat) and limit it to a maximum of 1 h for those aged 2 to 5 years, promoting alternative activities. It is emphasized not to use screens to pacify children, especially during meals and before bedtime. Healthcare providers play a crucial role in providing information to parents about the impacts of screen use and assisting in the creation of personalized media plans (20). The media industry is urged to produce interactive and age-appropriate content without advertising (unhealthy commodity industries such as tobacco, alcohol, and ultra-processed foods), and policymakers are called upon to collaborate on policies ensuring the safe development of young children exposed to screens (21).

Digital tools, when used strategically, can also support broader early childhood development initiatives. For example, in Brazil’s Programa Criança Feliz (PCF), WhatsApp was used to support program scale-up by facilitating rapid communication, networking, and capacity building. It helped share operational guidance, good practices, and updates, reaching even remote municipalities. This approach proved valuable in regions with political opposition, aiding coordination and accelerating adoption, demonstrating the potential of digital tools in implementing large-scale programs (6).

Similar to the role of digital tools in Brazil, studies from other developing countries have explored various facilitators and barriers to effective parenting. Studies from South Africa and Kenya highlight several factors that support positive parenting, such as strong social networks (family, partner, and community), healthy behaviors/environment, job opportunities, religion, information/knowledge, and professional assistance. Barriers encompassed low socio-economic circumstances (unemployment and financial difficulties), unsafe environments, lack of partner support, negative impact of technology, inadequate preparation for parenthood, and lack of access to services (16, 17, 22). Another study in Turkey emphasizes integrating early learning components into prenatal education programs for parents. These studies underscore the complexity of early childhood development challenges in the Nurturing Care Framework and the need for context-specific interventions (15).

In the theme of “The Need for Knowledge,” the study aligns with the WHO framework’s emphasis on knowledge and learning but reveals a significant gap in training and understanding among healthcare professionals, childcare workers, and community leaders, emphasizing the need for capacity development initiatives in line with WHO principles.

For the knowledge enhancement of healthcare and childcare professionals, significant challenges may exist, such as resistance to a long-term outcome approach, the need for a shift in training paradigms, hierarchical leadership challenges, and the importance of continuous professional education. The crucial connection between the effectiveness of Nurturing Care intervention and the healthcare system in which it operates is the main lesson for behavior change in healthcare professionals. Strengthening the system, with insights from organizational psychologists and healthcare professionals, is a necessary component of effective scaling techniques. Encouraging internal stakeholders to co-design and co-lead through a partnership approach promotes ownership and participation. Instead of creating more structures, the focus should be on improving current supervision and training. It is imperative to have a theory of change model that clearly describes the necessary steps for successful integration. Since adopting a new behavior is as important as learning a new skill, a realistic evaluation of the capacities and personnel of the healthcare system is essential (23).

The study acknowledges a relatively small sample size, particularly in the qualitative research paradigm, which could limit the generalizability of findings to broader populations in the Ecuadorian Highlands and beyond. It is crucial to recognize that the experiences and perceptions of participants may not fully represent the diversity within these communities, and the study may not capture the diversity of socioeconomic backgrounds within the chosen communities. Because snowball sampling was employed, it is possible that there was homogeneity in the data. Our chain referral asked seed participants to offer potential participants who would represent the diversity of the community, but this may not have been attained. Different socioeconomic groups may exhibit distinct nurturing care practices, and the study’s findings may not fully represent the entire spectrum. The interviews were conducted in Spanish, which is appropriate for the context but may exclude perspectives from individuals who speak other languages or dialects within the studied regions. Finally, although our research team is familiar with these communities and has performed substantial community-engaged research with them, no member of the team is native to the communities under study. There may be nuances of meaning that the researchers are unfamiliar with that an “insider” perspective could reveal.

The study provides nuanced insights into how nurturing care is perceived and practiced within the specific contexts of Sigchos and Calvas, Ecuador. This contextual understanding is crucial, adding depth to the global discourse on nurturing care, which often lacks granularity concerning diverse cultural settings. The study underscores the urgent need for training among healthcare professionals, childcare workers, and community leaders regarding nurturing care. Future research could focus on designing and evaluating training programs tailored to the specific needs and contexts of different stakeholders. The study mentions the preference for traditional medicine in certain situations, and future research could explore the integration of traditional practices with modern healthcare approaches, fostering a more holistic and culturally sensitive nurturing care strategy. While the study is specific to the Ecuadorian Highlands, the discussion effectively draws connections to broader global health challenges, contributing to the global discourse on nurturing care.

In conclusion, while the WHO’s Nurturing Care Framework provides a solid foundation, our study emphasizes the importance of cultural sensitivity, local contextualization, and targeted training initiatives to bridge gaps in understanding and application at the community level. The findings affirm the relevance of nurturing care principles and emphasize the need for nuanced, context-specific approaches to its implementation.

## Data Availability

The raw data supporting the conclusions of this article will be made available by the authors, without undue reservation.
